# Spin-Coated Heterogenous Stacked Electrodes for Performance Enhancement in CMOS-Compatible On-Chip Microsupercapacitors

**DOI:** 10.1021/acsaem.1c03745

**Published:** 2022-03-24

**Authors:** Agin Vyas, Simin Zare Hajibagher, Ulises Méndez-Romero, Shameel Thurakkal, Qi Li, Mazharul Haque, R. K. Azega, Ergang Wang, Xiaoyan Zhang, Per Lundgren, Peter Enoksson, Anderson Smith

**Affiliations:** † Department of Microtechnology and Nanoscience (MC2), Chalmers University of Technology, Kemivägen 9, 41296, Gothenburg, Sweden; ‡ Department of Chemistry and Chemical Engineering, Chalmers University of Technology, Kemigården 4, 41296, Gothenburg, Sweden; ¶ Enoaviatech AB, 112 26 Stockholm, Sweden; § Department of Electrical Engineering, Chalmers University of Technology, Hörsalsvägen 7, 41296, Gothenburg, Sweden

**Keywords:** microsupercapacitors, graphene, CMOS, MEMS, spin coated, stacking, modular

## Abstract

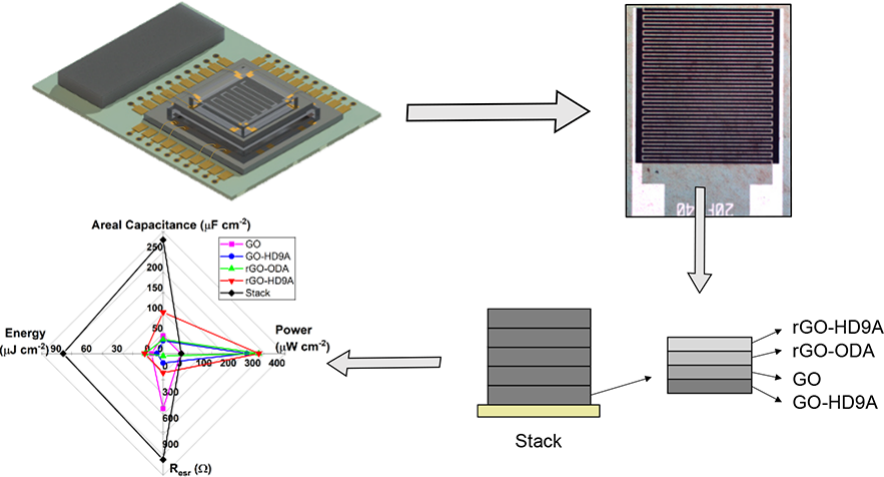

Integration of microsupercapacitors (MSCs) with on-chip sensors and actuators with nanoenergy harvesters can improve the lifetime of wireless sensor nodes in an Internet-of-Things (IoT) architecture. However, to be easy to integrate with such harvester technology, MSCs should be fabricated through a complementary-metal-oxide-semiconductor (CMOS) compatible technology, ubiquitous in electrode choice with the capability of heterogeneous stacking of electrodes for modulation in properties driven by application requirements. In this article, we address both these issues through fabrication of multielectrode modular, high energy density microsupercapacitors (MSC) containing reduced graphene oxide (GO), GO-heptadecane-9-amine (GO-HD9A), rGO-octadecylamine (rGO-ODA), and rGO-heptadecane-9-amine (rGO-HD9A) that stack through a scalable, CMOS compatible, high-wafer-yield spin-coating process. Furthermore, we compare the performance of the stack with individual electrode MSCs fabricated through the same process. The individual electrodes, in the presence of 1-ethyl-3-methylimidazolium bis(trifluoromethylsulfony)imide (EMIM-TFSI), demonstrate a capacitance of 38, 30, 36, and 105 μF cm^–2^ at 20 mV s^–1^ whereas the fabricated stack of electrodes demonstrates a high capacitance of 280 μF cm^–2^ at 20 mV s^–1^ while retaining and enhancing the material-dependent capacitance, charge retention, and power density.

## Introduction

Rapid steps in integration of microelectromechanical systems (MEMS) and complementary-metal-oxide-semiconductor (CMOS) technology based integrated circuits (IC) have led to an exponentially increasing demand for electronics and, in particular, mobile electronics.^1^ As electronic devices continue to shrink in size, while having better performance and expanding functionality,^2^ their growing abundance rapidly increases the total power requirements, traditionally relying on batteries as their primary power supply.^3^ However, in spite of extensive research on new sustainable materials^4−6^ for battery electrodes, their lifetimes are considerably lower than the requirements for a self-reliant sensor power supply, a problem which can be solved by integrating an energy harvesting/storage system into the device. Such integration would allow the device to be potentially self-powering.

Harvesting systems have traditionally relied on mechanisms such as sunlight, contact, or vibration to provide electrical energy.^7^ To power microelectronic circuits such as wireless sensor nodes, the energy requirement typically ranges from 100 μW to 10 mW.^8^ Silicon photovoltaics have commercial products available that can generate power for entire households.^9^ They have a conversion efficiency of 15% for 10 mW cm^–2^ of solar power present in the surroundings. MEMS vibrational piezoelectric energy harvesters and triboelectric energy harvesters have also demonstrated a potential to be on-chip devices that can support a self-reliant power supply functionality^10,11^ with harvesting capabilities of 4–100 μW cm^–2^. These devices are traditionally manufactured through CMOS compatible fabrication processes. A microsupercapacitor (MSC) is an ideal candidate for an energy storage unit for self-powering systems as it has the potential for a cycle life far superior to that of current state-of-the-art batteries.^12^ Furthermore, the high-power density of the MSCs is well suited for rapid charge/discharge cycles typical for a nanoenergy harvesters. However, harnessing the full potential of these technologies requires integration of MSCs through CMOS compatible technology for a seamlessly integrated self-reliant sensor node.

CMOS compatible processes are limited to certain selection of fabrication technologies such as sputtering, thermal annealing, physical and chemical vapor deposition, electron beam evaporation, photolithography, doping, and reactive ion etching. Devices that can be fabricated through CMOS compatible processes are viable for on-chip integration with integrated circuits.^13^ To integrate MSCs into CMOS and MEMS technology in a front-end-of-line (FEOL) process, we need to address two major needs of integration. First, they must be fabricated through scalable methodologies compatible with CMOS and MEMS technology and second, the process must be ubiquitous for deposition of electrode material engineered for high energy, low self-discharge, or any other special characteristics tailor-synthesized to fulfill application necessities.

A literature survey addressing the needs of scalability and MEMS compatibility shows that silicon substrate based MSCs can be fabricated through CMOS compatible technologies such as chemical vapor deposition,^14^ laser scribing,^15^ inkjet printing,^16^ doping,^17^ layer-by-layer assembly,^18^ pyrolysis,^19^ and spin coating.^20,21^ Furthermore, several scalable techniques such as vacuum filtration,^22^ xurography,^23^ spray coating,^24^ and sputtering^25^ have been applied for fabrication of solid state and flexible MSCs. Among all these processes, spin coating is one process that can be considered truly material versatile while being CMOS compatible. Spin coating is not particularly dependent on material choice; several electrode materials such as rGO,^26^ carbon nanotubes (CNTs),^15^ carbon nanofibers (CNFs),^14^ onion-like carbon (OLC),^27^ carbide-derived carbon (CDC),^28^ graphene nanoparticles (GNPs),^29^ and exfoliated graphene^30^ can be synthesized into aqueous organic/inorganic solutions; these are critical for good spin coating.^31^ Among these, rGO has demonstrated a capacity for high-energy and -power density MSC, with graphene with large specific surface area (2630 m^2^ g^–1^). Furthermore, rGO can be combined with several functionalization agents to improve the capability of MSCs in the above-mentioned terms.^32^ Spin-coated MSCs geared toward CMOS compatibility have been fabricated by Huang et al.^33^ through laser cutting patterns on a Kapton tape mask on a Si substrate. The material is spin coated on the exposed surfaces to fabricate interdigitated electrodes. Similarly, Zhang et al.^34^ have also demonstrated fabrication of graphene-based solid state MSC through spin coating. Both these processes, however, use Kapton tape as a masking agent. Recently, graphene-based composites mixed with Ag nanowires or single walled CNTs have been used as spin-coating material for plotter-assisted fabrication of all solid MSCs.^23^ Similarly, graphene has been used with a combination of MXenes with CNTs for low pass filter application.^35^ Composites of 3D-laser-induced graphene foams mixed with ZnP nanosheets have also been used for integration with triboelectric nanogenerators (TENG) in a scalable fabrication process through spin coating.^36^ Another set of spin-coated MSCs were fabricated by Mai et al.,^37^ where the electrodes were composed of alternated stack of rGO–CNT photoresist composites. The MSCs further illustrated spin-coating with graphene as base electrode material for property modulation as a perfect candidate for a low cost, high energy density MSC fabrication process also compatible for integration with microenergy harvesting techniques. However, to be integrated in applications such as the IoT, body implants, and RFID systems, the fabrication of MSCs through spin coating requires a standardized process plan whose process steps are incorporable in a current CMOS/MEMS fabrication facility.

We have previously demonstrated a spin-coating process for the fabrication of MSCs in a CMOS compatible spin-coating process by fabricating graphene-based rGO electrodes through an aluminum hard mask.^38^ These devices however suffered from issues with electrode adhesion, wafer coverage of material, and uniformity in thickness of spin-coated electrodes. The spin-coating process was improved by utilizing surface roughening through Fe nanoparticles for enhanced coverage, adhesion, and uniformity.^21^ However, until now, this process has not been tested for stacking a variety of electrode materials to yield modular properties such as optimizing for energy density while demonstrating good power density.

Thus, this article demonstrates the fabrication of a modular composite stack of functionalized rGO based on four different materials. The electrode materials include postannealed reduced graphene oxide (GO), postannealed branched-alkylamino GO-heptadecane-9-amine (GO-HD9A), prereduced linear rGO-octadecylamine (rGO-ODA), and prereduced rGO-heptadecane-9-amine (rGO-HD9A), spin-coated layer-by-layer in a bottom-up approach. Organic groups such as heptadecane-9-amine (HD9A) and octadecylamine (ODA) chains improve rGO’s solution stability^39^ while preserving its latent characteristics. HD9A and ODA are branched and linear alkylamine chains, respectively. These functional groups can also allow rGO to be combined with several different materials in order to modulate the MSC characteristics. This stack of individual rGO based materials have an additive response to the capacitance characteristics of the Stack-MSC. The critical evaluation of singular-material MSCs has also been performed for each constituent of the electrode stack. We show that the MSC containing the stack of electrode materials conforms to the properties of the MSCs fabricated with individual constituent elements. The MSCs -GO-, GO-HD9A, rGO-ODA, and rGO-HD9A in the presence of 1-ethyl-3-methylimidazolium bis(trifluoromethylsulfony)imide (EMIM-TFSI), demonstrate a capacitance of 38, 30, 36, and 105 μF cm^–2^ at 20 mV s^–1^ respectively; the fabricated stack (Stack-MSC) demonstrates a capacitance of 280 μF cm^–2^ at 20 mV s^–1^ while retaining and enhancing material dependent capacitance, charge retention, and power density.

## Fabrication of MSCs

**Figure 1:**
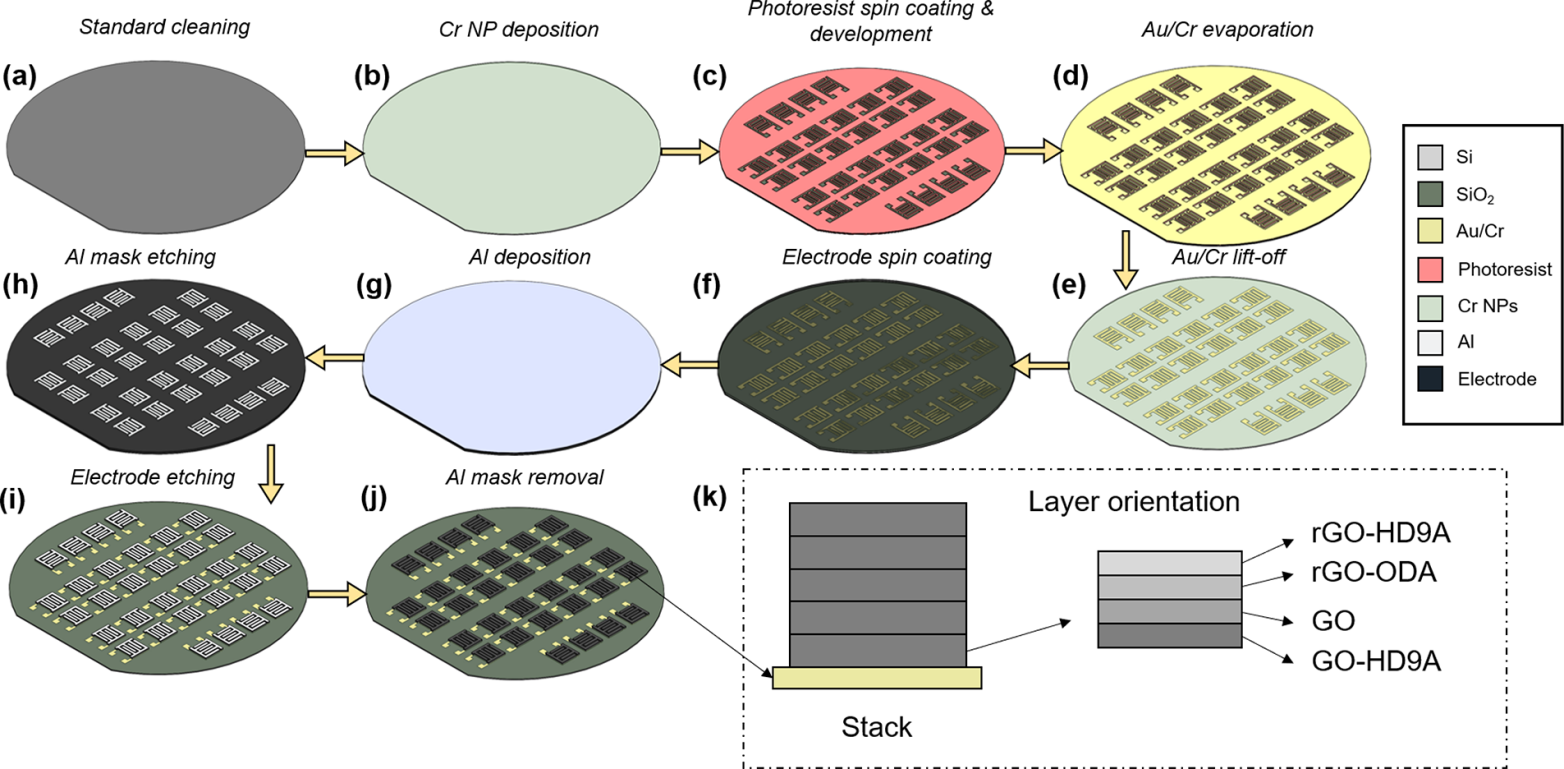
(a–j) Schematic MSC fabrication process through spin coating of electrode solution and subsequent hard masking via an aluminum layer. (k) Schematic orientation of Stack MSC electrodes during the spin-coated deposition process step.

The MSCs with stacked electrode materials are fabricated on a 2-in. Si substrate (Figure 1a). The substrate is cleaned in 1:1 NH_3_:H_2_O_2_ and 1:1 HCl:H_2_O_2_ at 80 °C for 10 min, respectively. A 400 nm SiO_2_ layer is thermally grown on the substrate surface to make the devices insulating. The SiO_2_ surface is roughened through a thin Cr (2 nm) film annealed at 500 °C^21^ (Figure 1b). Au/Cr (100/20 nm) current collectors are deposited on the surface through a lift-off photoresist mask. The photoresist, LOR-3A (Microchem) and S1813 (MicroChem) is spin coated on the surface roughened substrate at 4000 pm and 2000 rpm s^–1^ (Figure 1c). Soft baking at 180 °C for 5 min and 120 °C at 1 min 20 s is conducted. The spin-coated photoresist is exposed in ultraviolet mask aligner under a custom chromium hard mask with current collector patterns. The photoresist is developed in MF-319 developer for 90 s. The substrate is descummed in O_2_ for 1 min at 100 W. Later, gold and titanium are evaporated on the surface-enhanced substrate through electron beam evaporation (Figure 1d). The lift-off photoresist was developed in mr-REM 400 (MicroChem) remover solution at 35 kHz sonication for 55 min. Once the current collectors are fabricated (Figure 1e), we spin coat the electrode solutions at varying spin speeds acquired through rigorous testing of spinning all the solutions at different spin speeds to acquire the most uniform coverage on a Cr covered Si chip. For GO, GO-HD9A, rGO-ODA, and rGO-HD9A-MSCs, five layers of each were spin coated, while in the stacked MSC, all the four layers of different material are spin coated five times between intervals of vacuum drying at 100 °C (Figure 1f). The resultant thickness achieved for each layer is 180 ± 20, 130 ± 20, 170 ± 20, 595 ± 40, and 1.2 ± 0.1 μm for GO, GO-HD9A, rGO-ODA, rGO-HD9A, and Stack-MSCs, respectively. The representation of layer order in Stack MSC is shown in Figure 1k. Once the layers have dried, poly(methyl methacrylate) (PMMA) polymer coating (20 nm) was spin coated on the surface to improve surface uniformity. An Al layer of 200 nm is deposited through electron beam evaporation (Figure 1g) on the surface of the polymer and then etched in the gaps of the MSC pattern through reactive ion etching via a Cl_2_/SiCl_4_ mixture in the presence of Ar (Figure 1h). The electrode stack was then etched away using an O_2_ plasma recipe at 100 W at 100 mTorr (Figure 1i). Finally, the Al hard mask is etched and the samples are annealed at 500 °C to convert GO to rGO for improving the device resistance by uniformizing the stack^40^ (Figure 1j).

**Figure 2:**
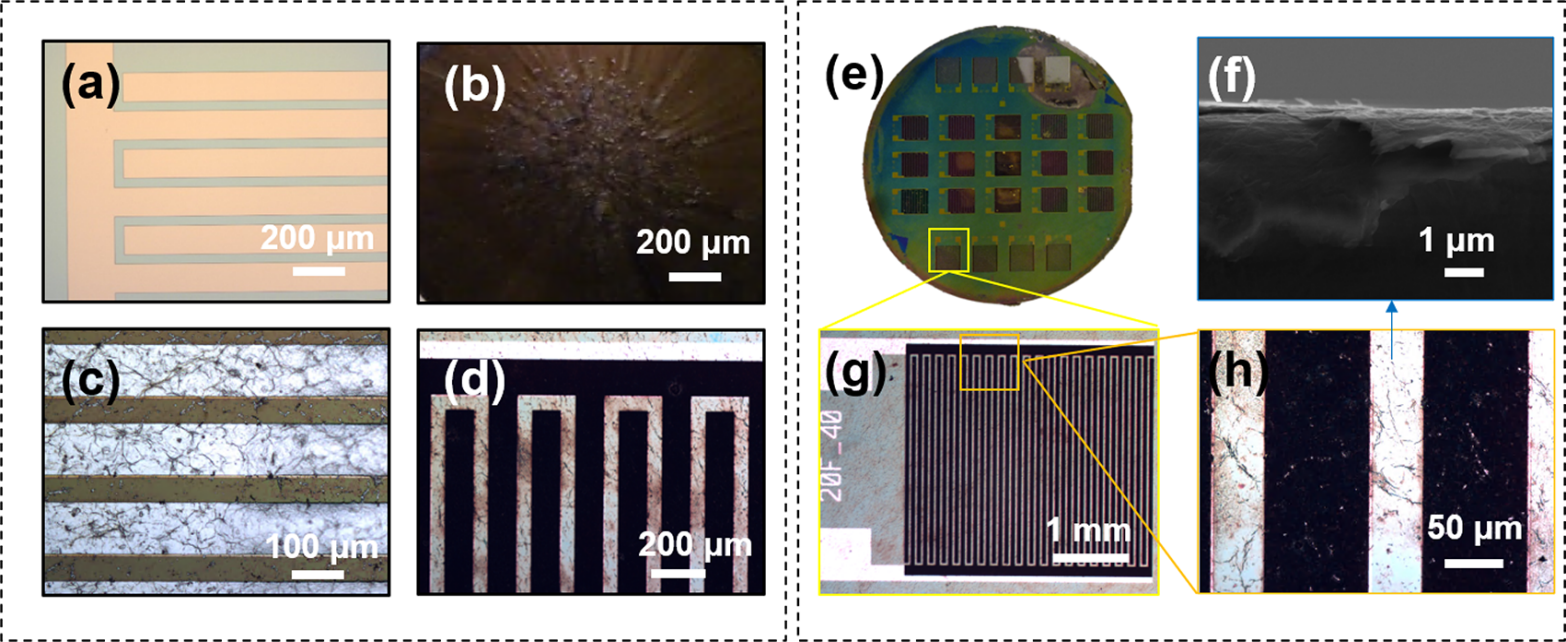
(a) Au/Cr current collectors post lift-off.(b) Spin coated mixture of solutions on the wafer substrate.(c) Aluminum hard mask.(d) Stack electrodes on current collectors at the final step. (e) Camera capture of the fabricated Stack-MSC wafer (2 in.). (f) Cross section of the fabricated MSC with conductive rGO layers of approximately 1.1 μm. (g) Optical micrograph of the Stack-MSC. (h) High resolution optical image of the finger geometry of the Stack-MSC post GO reduction.

Parts a–d of Figure 2 show the optical micrographs of the wafer substrate after each respective fabrication step. As visible in the images, the process is conformal to photoresist development with the final electrode stack taking the exact form in Figure 2d of the current collectors shown in Figure 2a. This demonstrates the feasibility of taking the device design down to micrometers, even nanometers if the lithography process enables such resolution. The wafer yield can be seen in Figure 2e, where 18 out of 24 devices fabricated on the wafer are functional. Observations from cross sectional SEM micrographs suggest a stacking of rGO sheets over a planar area in Figure 2f. The optical micrographs in parts g and h of Figure 2 show that the interdigitated fingers do not have any unetched material that can act as shorts.

## Results

In this section, we will first discuss the performance of individual electrodes based MSCs, i.e., GO-MSC, GO-HD9A, rGO-ODA, rGO-HD9A and then describe the electrochemical performance of the Stack-MSC, all fabricated through the process described in the Fabrication of MSCs. Finally, we present a comparison of Stack-MSC with its constituent MSCs.

**Figure 3:**
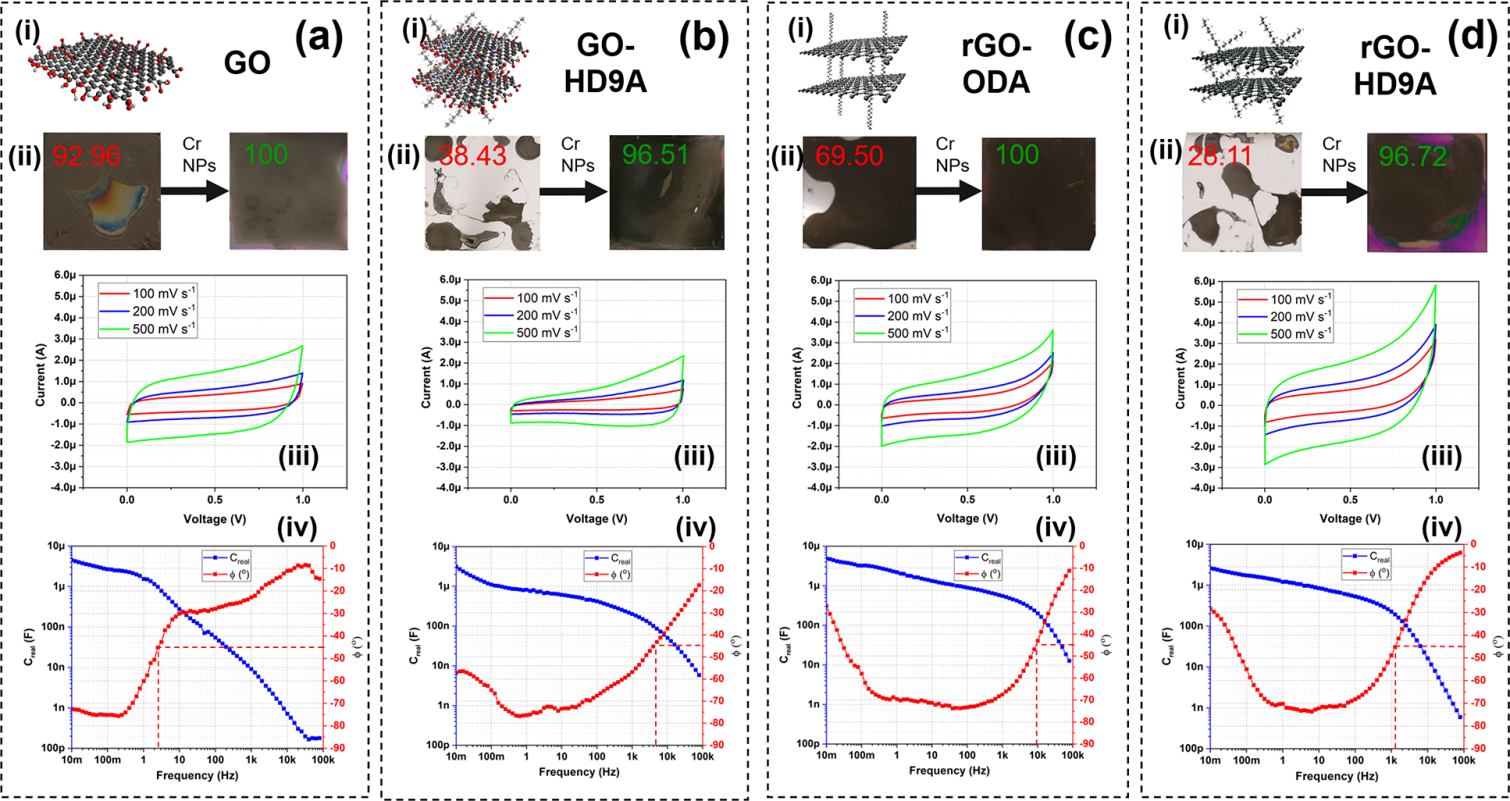
(i) Schematic representation of the atomic configuration. (ii) Improvement in coverage of spin-coated electrodes after addition of a Cr nanoparticle layer (experiments conducted on a 1 cm^2^ Si chips). (iii) cyclic voltammograms of MSC with 20 fingers with 40 μm spacing fabricated by process described in 1 at different scan rates ranging from 100–500 mV s^–1^. (iv) Bode plot for fabricated MSCs, for 3 g/L (a) GO in H_2_O (reduced postprocess), (b) GO-HD9A in ODCB (reduced postprocess), (c) rGO-ODA in ODCB, and (d) rGO-HD9A in ODCB.

The effects of surface roughening on spin-coating and performance of individual MSCs, GO, GO-HD9A, rGO-ODA, and rGO-HD9A are shown in Figure 3a–d. In Figure 3i, the molecular structures of different materials are outlined. The GO layer in Figure 3a–i is a layer of graphene with various oxidation sites, marked as red. GO-HD9A, similarly, is graphene functionalized with a branched alkane with an amine functional group, heptadecane-9-amine, and specific bonding sites. This functionalization has been achieved to increase the surface adhesion of GO and to facilitate thickness uniformity. rGO-ODA is a similar functionalization with a linear alkane group instead of branched with amine end. Moreover, it is also prefunctionalized in the solution and thus does not require post-process annealing for GO reduction. Finally, rGO-HD9A is prereduced GO-HD9A with similar properties as its precursor. The effects of surface roughening on the electrode solutions can be observed in Figure 3ii, more specifically for functionalized GO solutions where we see an extremely high change in surface coverage compared to nonroughened substrates. The electrochemical results of the fabricated MSCs can be seen in Figure 3iii. At first, it is not intuitive to see that rGO-HD9A holding the highest areal capacitance, as we would expect rGO-ODA to demonstrate a higher specific surface area due to its unbranched alkane chain. However, since the amount of deposited rGO-HD9A, increased through higher steric interaction, is substantially higher than for the other materials, we see a higher capacity to store charge. The steric hindrance of the molecules increases the area of interaction for the subsequent spin coating of layers. The amount of deposited rGO-HD9A is approximately 3 times higher than that for GO, GO-HD9A, and rGO-ODA. However, on normalization of capacitance with respect to device volume, we notice that rGO-HD9A is among the worst performing materials. This too can be attributed to the presence of steric hindrance within the molecules.

GO-MSC in Figure 3a-iii shows a stronger quasi-rectangular behavior (determined by *R*) compared to other materials. *R* is the ratio to an ideal capacitor with a rectangular cyclic voltammogram. This can be attributed to the post annealing step that removes all the impurities from the GO flakes while reducing GO to rGO. Similarly, we see the equivalent response for GO-HD9A in Figure 3b-iii. In Figure 3iv, we evaluate the EIS spectra of the individual materials. As visible, postprocess GO demonstrates a stronger resistive behavior due to issues related to vertically oriented electrical conductivity of the film. GO-HD9A, rGO-ODA, and rGO-HD9A improve the frequency behavior of the MSC substantially through their modulated structural van der Waals bonding in the alkane chains. GO-HD9A does not store a large amount of charge, however, it shows the lowest *R*_*esr*_ among the fabricated MSCs while demonstrating the highest uniformity in performance over a large number of tested MSCs in the same batch. In summary, the chosen four materials had unique properties such as GO for strong EDL behavior, GO-HD9A for uniformity, rGO-ODA for capacitance improvement and rGO-HD9A for improved thickness.

**Figure 4:**
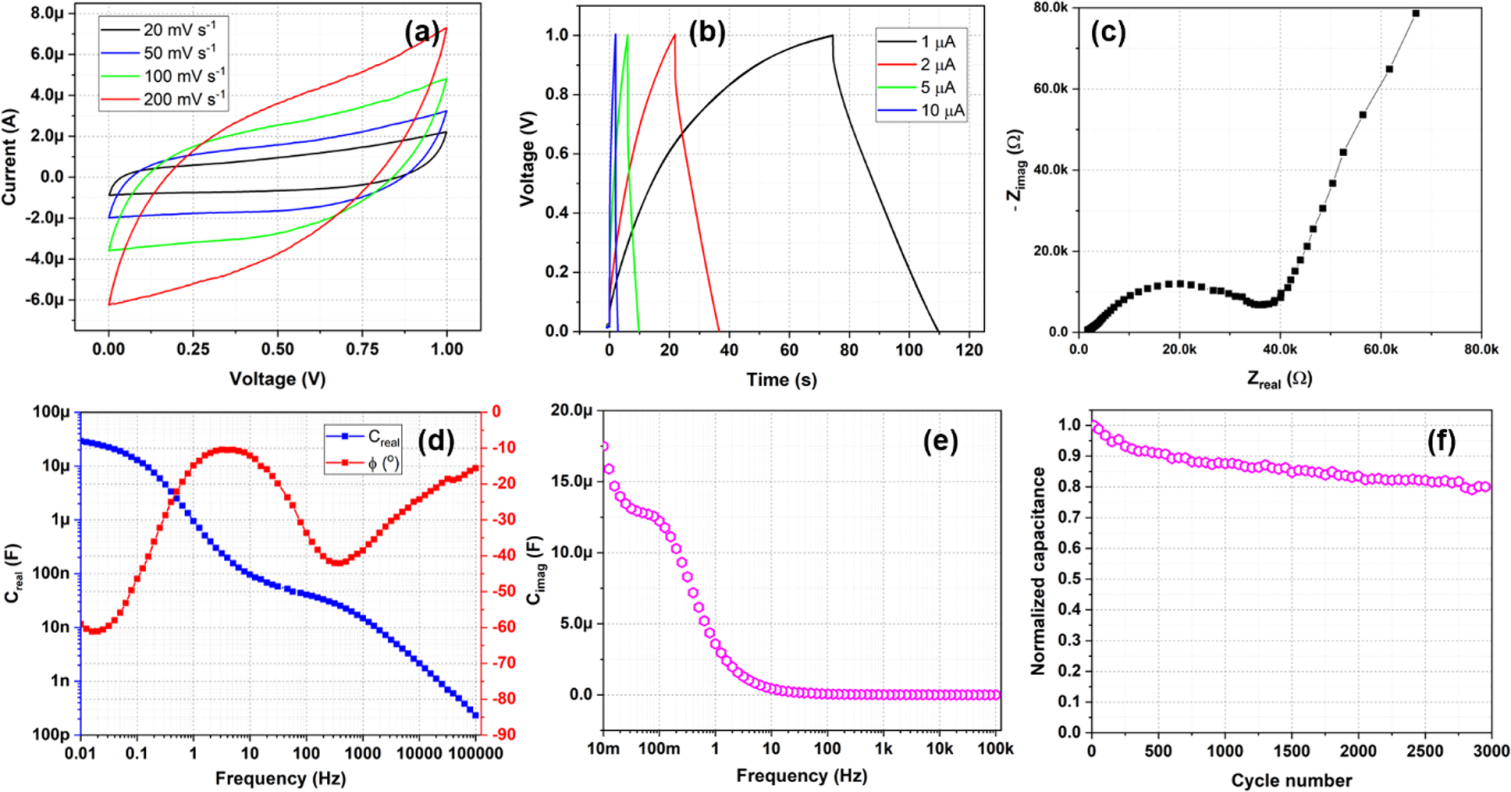
Electrochemical characterization of Stack-MSC: (a) Cyclic voltammetry at various scan-rates and (b) GCD measurement. EIS performance of the Stack-MSC represented as (c) Nyquist, (d) Bode with *C*_*real*_ and ϕ as functions of frequency, and (e) Bode in terms of *C*_*imag*_ over a range of operational frequencies. (f) Normalized capacitance of Stack-MSC over several cyclic charge–discharge cycles.

The MSC stack constituted a layer of GO-HD9A at the bottom to improve uniformity, followed by GO for strong EDL behavior, followed by rGO-ODA for vertical conductivity and rGO-HD9A for higher electrode deposition and adhesion improvement during post processing steps. A further reason for the particular choice of material stack was obtained through EIS analysis of the fabricated MSCs. The stack design is based on a layer by layer approach of depositing material with the highest conductance first, followed by subsequent layers, i.e., GO-HD9A > GO > rGO-ODA > rGO-HD9A. The high parasitic resistance of prereduced MSCs can be attributed to the presence of amine groups and their tail ends interacting with EMIM-TFSI electrolyte, causing higher charge transfer resistance.^41^ The chosen materials were spin-coated in a quasi layer-by-layer process in the order GO-HD9A, GO, rGO-ODA, and rGO-HD9A, from bottom-to-top. The stacking process was repeated five times to achieve the fabricated Stack-MSC. It is important to note that the stacking was not just four layers in total with one material per layer, but a mixed stack with five layers of heterogeneous stacks. Figure 4 shows the electrochemical performance the Stack-MSC at various input signals.

In Figure 4b, it can be noted that the Stack-MSC demonstrates a longer time for charging at 1 μA cm^–2^ leading to an increased curvature in the GCD cycle. This shows that the leakage current influences the rate of MSC charging. Figure 4c shows the Nyquist plot of the MSC as a function of *Z*_*real*_ and −*Z*_*imag*_ obtained from eqs 4 and 5 (Section S3, Device Characterization, Supporting Information: equations for real and imaginary capacitance). The device reveals an equivalent series resistance (*R*_*esr*_) of 1.2 kΩ. This can be corroborated with the IR drop from the GCD measurements. The charge-transfer resistance of the Stack-MSC is larger as the transfer resistance of each material adds on successively in a serial combination. In-spite of its high resistance, the power density of the device is equivalent to the power density observed in GO-MSC. The Warburg impedance observed through the slanted line (after semicircular charge-transfer resistance) is indicative of a porous electrode medium. The real capacitance (*C*_*real*_) and phase angle (ϕ) from eq 7 (Section S3, Device Characterization, Supporting Information: phase response of the output) are shown in Figure 4d, while *C*_*imag*_ is shown in Figure 4e. Here, we can see that as the frequency increases above 10 Hz, the *C*_*imag*_ component of Stack-MSC tends to zero, giving rise to a highly resistive device behavior. Finally, in Figure 4f, we see the cyclic charge–discharge nature of the Stack-MSC over 3000 cycles of charging and discharging at 5 μA cm^–2^. As we can see, the device demonstrates 81% of its maximum capacitance at this specific current density after only 3k cycles. The performance is however comparable to several state-of-the-art MSCs presented in literature.^42^

**Figure 5:**
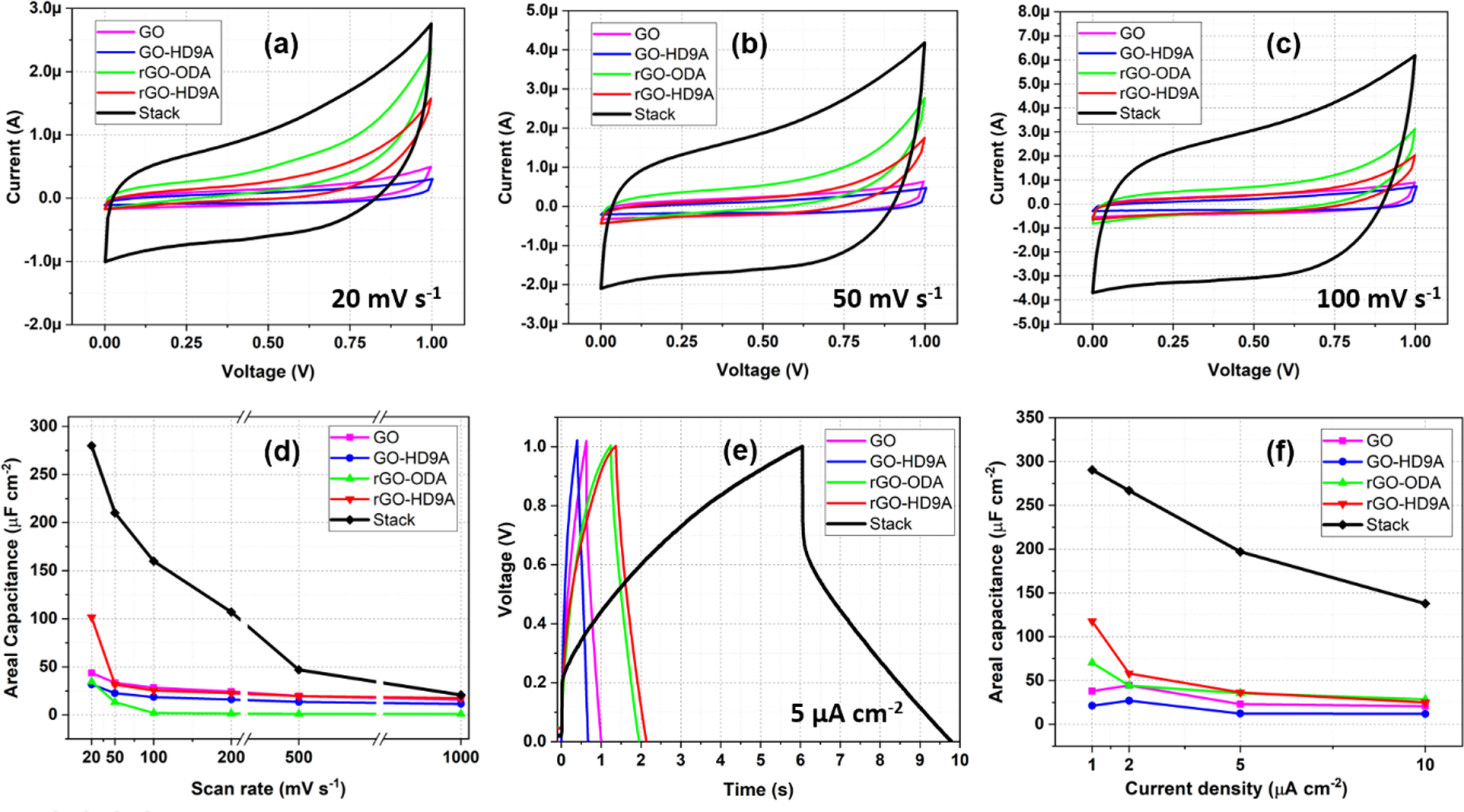
(a–c) Cyclic voltammograms of the four materials, GO, GO-HD9A, rGO-ODA, and rGO-HD9A-MSCs. in comparison to Stack-MSC at (a) 20, (b) 50, and (c) 100 mV s^–1^. (d) Comparison of areal capacitance of fabricated MSCs over a range of scan-rates, (e) chronopotentiometric scan of MSCs at 5 μA cm^–2^, and (f) areal capacitance of the MSCs when charge–discharged at various current densities.

The performance of Stack-MSC in comparison to individual material MSCs namely GO-, GO-HD9A-, rGO-ODA-, and rGO-HD9A-MSCs is shown in Figure 5. Parts a–c of Figure 5 show the cyclic voltammograms of the spin-coated MSCs at 20, 50, and 100 mV s^–1^. At 20 mV s^–1^ charging, the behavior for rGO- and HD9A-MSCs appears to be quasi rectangular in shape. However, rGO-ODA- and rGO-HD9A-MSCs show a resistive behavior while scanning toward potentials over 0.5 V. This behavior is inherited by the Stack-MSC. Another interesting feature is that the sum of the capacitances of single electrode material MSCs is 361 μF cm^–2^ while the Stack-MSC displays an areal capacitance of 307 μF cm^–2^. The slight acuteness can be attributed to material loading at specific sites on the spin-coated wafer. Figure 5d further elucidates the finding by showing the trends for areal capacitance of different fabricated MSCs over increasing scan-rates. At 1000 mV s^–1^, the areal capacitance of the Stack-MSC is comparable to single material MSCs. Figure 5e shows GCD measurements of MSCs at 5 μA cm^–2^. As seen also in Figure 4b, the Stack-MSC has a larger *R*_*esr*_ compared to other material MSCs. Figure 5f shows the areal capacitance of MSCs at varying current densities. As visible, at higher current density of 10 μA cm^–2^, the capacitance retention for Stack-MSC is about 50% of its original capacitance. The large drop in areal capacitance at higher current densities can be attributed to presence of the high parasitic resistance derived from the material’s internal resistance. Also, the current supplied to the MSCs is calculated using the nominal total device area of 1 cm^2^. As the actual MSC electrode area is only 0.11 cm^2^, the current density in the electrode should be scaled up to 2 orders of magnitude accordingly.

**Figure 6:**
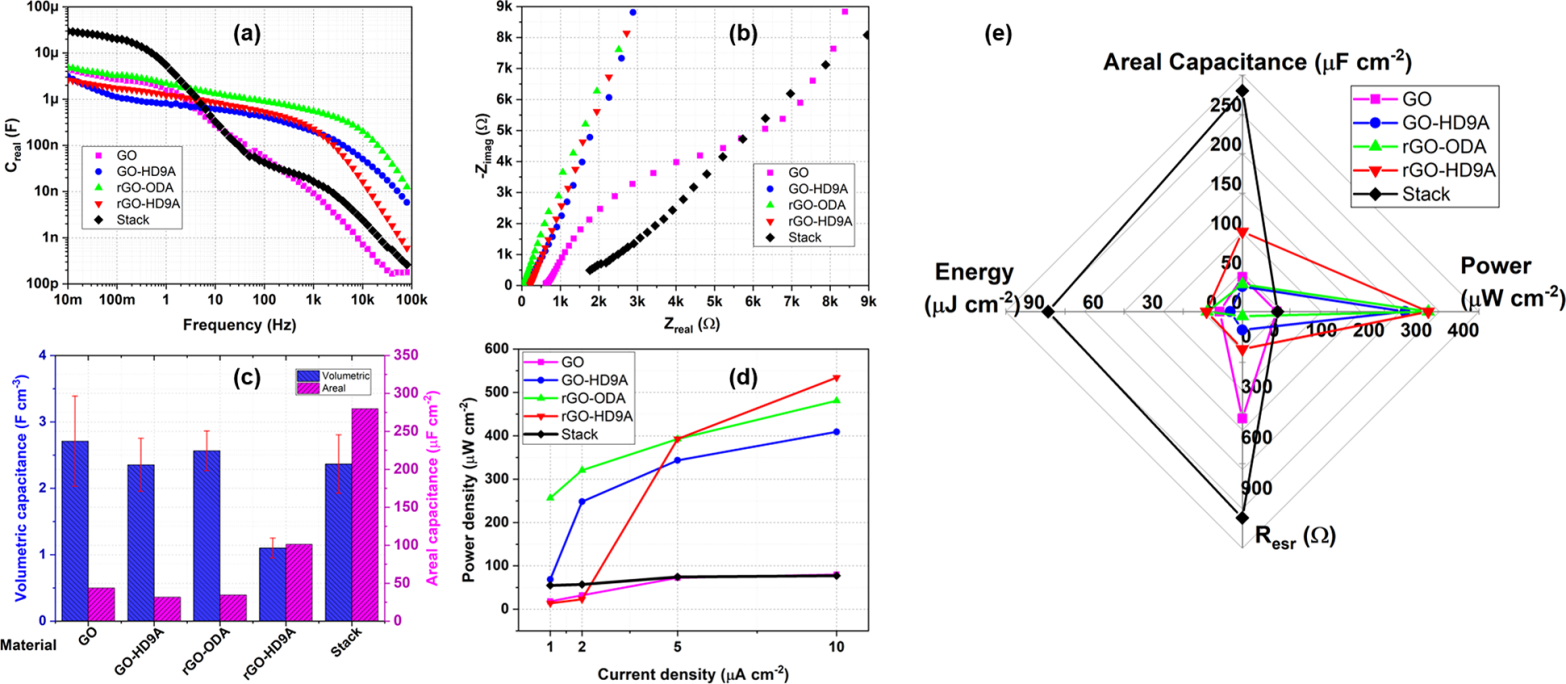
(a) Bode plot of *C*_*real*_ vs frequency for Stack, GO, GO-HD9A, rGO-ODA, and rGO-HD9A. (b) Nyquist plot of the fabricated MSCs. (c) Comparison of volumetric capacitance and areal capacitance of individual material with Stack. (d) Areal power density of MSCs over increasing current density. (e) Ragone plot of energy and power density of the MSCs fabricated in the current manuscript in comparison to MSCs with EDLC material published in the recent literature.

Figure 6a shows the *C*_*real*_ for MSCs calculated from eq 4 and 5 (Section S3, Device Characterization, Supporting Information: equations for real and imaginary capacitance) from the EIS measurements. This form of Bode plot is useful in evaluating the confluence of capacitive and resistive device behavior through *C*_*real*_ trends. At frequencies higher than 100 Hz, the capacitive performance appears to track the strong *C*_*real*_ traits of rGO-ODA- and rGO-HD9A-MSCs. The behavior of Stack-MSC is strongly affected by the slowest response in the stack. Figure 6b shows the Nyquist plot of the fabricated MSCs. Here, we can clearly see a trend of series resistance from the materials in the Stack-MSC. The total resistance of individual MSCs’ was *R*_*esr*_ = 975 Ω while the Stack-MSC has an *R*_*esr*_ = 1.2 kΩ. GO-HD9A-, rGO-ODA-, and rGO-HD9A-MSCs show a porous electrode medium with their Nyquist slopes tending more toward the ideal capacitor behavior, while the GO-MSC reveals a high charge-transfer resistance. The Stack-MSC also shows a high charge-transfer resistance, which further strengthens the inference that individual material properties can be directly determining the properties of the combined stack.

After analyzing the material performance further, we evaluate the individual volumetric capacitance of the MSCs alongside their areal capacitances at 20 mV s^–1^ in Figure 6c through information on the electrode thickness acquired from surface profiler measurements. The error bars correspond to standard deviation observed during thickness measurement. GO as a material demonstrates the highest volumetric capacitance followed by rGO-ODA, GO-HD9A, and then rGO-HD9A. The stack material reveals a volumetric capacitance comparable to other electrodes, so it can be assumed that the material has not undergone any severe transformation during spin coating of the stack. The average volumetric capacitance of the combined materials is 1.7 F cm^–3^ compared to volumetric capacitance of the stack as 2.3 F cm^–3^. Both the metrics are equivalent within the measured accuracy range. Figure 6d shows the power density and areal capacitance of the MSCs over a range of current densities. The material MSCs individually manage to deliver higher power densities at larger currents, especially GO-HD9A-, rGO-ODA-, and rGO-HD9A-MSCs. The resistive nature of Stack and GO leads to a poor power density for these devices. Finally, in Figure 6e, we compare the performance of the MSCs fabricated as a plot displaying their individual energy, power, capacitance densities, and equivalent series resistance.

## Discussion

Based on our analysis of the electrochemical results for surface enhanced spin-coating fabrication for Stack, GO, GO-HD9A, rGO-ODA, and rGO-HD9A MSCs, we present an equivalent circuit analysis for the devices in this section. The rationale for our material choices will also be discussed. Finally, we will compare the performance of the fabricated MSCs with devices in literature using either the same material or a combination of various related meta-materials.

**Figure 7:**
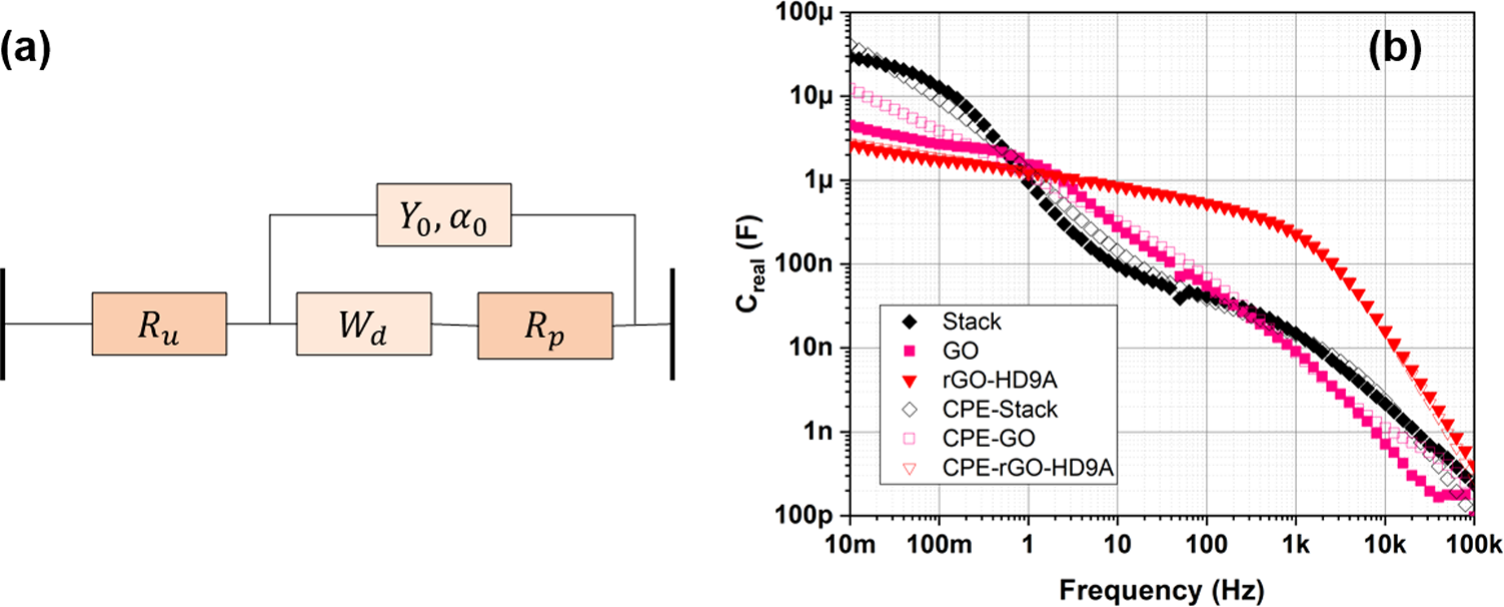
(a) Equivalent circuit CPE with diffusion model (Bisquert et al.^43^). (b) Analysis of the fabricated MSCs: Stack, GO, and rGO-HD9A. Table 1 shows the calculated values for different components for the equivalent circuit.

Figure 7a shows the EIS Bode spectrum of the *C*_*real*_ for the fabricated devices using the standard equivalent circuit model constant phase element with diffusion (CPE-diff). The CPE-diff model proposed by Bisquert et al.^43^ is considered as a standard referential tool in literature. The model has been explained in Huang et al.^44^ to model impedance responses in disordered mediums such as thick rGO films.^45^ In this circuit, *R*_*esr*_ is shown by *R*_*u*_ in series with a constant phase element  in parallel with the combination of a *R*_*p*_ (charge-transfer resistance) and a Warburg admittance  in series, where α_0_ is the variable that is representative of the electric response related to porosity. In Table 1, we see the resulting parameter values when fitting the model for the different devices—Stack, GO, GO-HD9A, rGO-ODA, and rGO-HD9A MSCs. After investigating the equivalent circuit parameter values for MSCs, we see that the net resulting *R*_*u*_ for the Stack is not far off the sum of the constituents’ *R*_*u*_ values (within 20%). Similarly, the stack demonstrates a correlation to four constant phase elements in series (*Y*_0_^α_0_^). Both these factors fit the explanation to the behavior of the stacked electrode. For the Stack-MSC, *W*_*d*_ is higher which shows its improved charge retention compared to individual materials. The Warburg admittance parameter also reflects the low frequency response where the stack shows a higher capacitive value. Similarly, *R*_*p*_ for the Stack-MSC is lower than all MSCs excluding GO-MSC, another important consideration for charge retention through lower leakage current. The major inference is that the Stack-MSC demonstrates a combination of capacitance for all the materials at low frequencies, but it is heavily influenced by the behavior of the material with smallest *C*_*real*_ capacitance, namely GO, in this case at high frequencies. The main advantage of the stacked-based fabrication technique is related to its versatility with regards to deposition of different materials. We have tested the deposition of a variety of pseudocapacitive electrodes through spin coating. However, during our trials, spin coating of such electrode materials on a substrate cannot experimentally lead to a good wafer yield. One possible alternative is to mix them in a solution of graphene-based material.

**Table 1:** Equivalent Circuit Modelling Results for the Fabricated MSCs

element	Stack	GO	GO-HD9A	RGO-ODA	RGO-HD9A	Error (±)	Units
*R*_*u*_	1221	633	109	28	221	25	Ω
*Y*_0_	0.4μ	3.7μ	1.3μ	3.2μ	1.7μ	19n	S* *s*^α^
α_0_	0.69	0.66	0.82	0.81	0.83	7m	–
*W*_*d*_	17μ	1.2p	0.82μ	0.71	0.23	0.2μ	S* *s*^0.5^
*R*_*p*_	27.4k	18.9k	0.7M	1.9M	2.9M	407	Ω

For the MSC fabrication process to be compatible with CMOS fabrication, MSCs need a process plan that can be incorporated within a MEMS or CMOS process scheme without affecting the material or design quality for the other devices. While keeping these constraints in mind, the MSCs discussed in this article are fabricated using conventional photolithography, electron beam evaporation, reactive ion etching, and low temperature annealing. MEMS and CMOS devices generally require several UV lithography steps for their fabrication. The process plan for the MSCs can be incorporated at any point in the MEMS process level sequence without requiring the wafers to be transported out of the cleanroom, any Kapton masking step or using tools such as focused ion beam for RIE, to count a few general practices applied in MSC fabrication in the recent literature.^46^ Furthermore, the issue of material loss during typical cleanroom processes such as photoresist development and ultrasonication has been mitigated through application of surface-roughened Cr NPs,^21^ as discussed in our previous publication.

There are a few issues regarding the CMOS compatibility of MSC fabrication that still needs to be addressed before the process can be implemented for integrated systems on-chip. First, graphene-based materials are often highly conductive flakes that can cause unwanted short circuit if they fly off and stick to other devices being fabricated in a FEOL process. Incorporation of pseudocapacitive materials such as MnO_2_, V_2_O_5_, Co_3_O_4_, and others with graphene-based inks can also potentially lead to similar problems. Therefore, further work must be conducted on issues related to flaking of bulk graphene layers during fabrication in low pressure and high temperature environments. Second, application of Au/Ti as current collectors is not an optimum solution from a compatibility perspective. Using Pd, Pt, Mo, W, or Cr as current collectors can also be interesting options for future work as these metals are considered CMOS compatible, and they demonstrate high conductance. However, when we fabricated MSCs using Pd/Ti as current collectors and vertically oriented carbon nanofibers as the electrode material, we observed a 10 times reduction in the device capacitance and a considerable increase in device resistance while using H_2_SO_4_/PVA as an electrolyte.^47^ Finally, using aqueous or gel electrolyte with CMOS circuit fabrication is not advisable, so there also needs to be a study on proper packaging solutions for such materials.

Increasing the number of spin-coated layers results in a higher thickness. By increasing the substrate surface roughness, we can achieve 1.1 μm thick rGO spin-coated electrodes as discussed in our previous publication.^21^ The capacitance of those devices was 110 μF cm^–2^ (comparable to rGO-HD9A in this study). These values for rGO were achieved employing Fe nanoparticles for surface roughening. Fe nanoparticles are not CMOS compatible as Fe’s boiling point is extremely low compared and furthermore, Fe is a reactive metal when in contact with ionic solutions. Therefore, in this study, we have used Cr nanoparticles which have a higher boiling point and lower reactivity, albeit with slightly lower surface roughness. Cr is a material also used in other CMOS compatible fabrication procedures.^48^

MSCs fabricated through spin coating show relatively low energy densities due to utilization of EDLC material only. In comparison to various silicon based MSCs which are fabricated through hard masking technique, the Stack-MSC performs comparatively well. Graphene-based MSC was fabricated by Wu et al.^49^ using graphene transfer and current collector hard mask as electrode deposition and etching process, respectively. The MSCs delivered a performance of 80 μF cm^–2^ with an electrode thickness of 15 nm. Similarly, Beidaghi et al. combined rGO with CNTs to produce MSCs through transportation of electrode ink in SU-8 channels. The resultant device yielded a capacitance of 6.1 mF cm^–2^ in the most optimum rGO–CNT composition. The high areal capacitance can be attributed to thicker electrodes deposited. Li et al.^50^ showed on-chip MSCs using MnO_2_ in porous carbon mixture. Graphene based electrode materials have been combined with various materials such as V_2_O_5_, MoS_2_, CNTs, and various EDLC or pseudocapacitive materials for higher energy densities. For example, Boruah et al.^24^ fabricated a sandwich-type MSC using mask assisted spray deposition of electrodes on a flexible substrate. As discussed previously, Yang et al.^19^ demonstrated a stack of MoS_2_, rGO, and CNT mixed with photoresist (pyrolyzed later) for extremely high-energy densities through spin-coating. GO has also been combined with CNT powder in laser-scribed flexible MSCs for higher energy densities as described by Wen et al.^15^ Laser scribing is a viable alternative to spin coating. However, the laser scribed devices, when combined with different materials show a large *R*_*u*_ because of the high-power discharge of lasers during GO to rGO conversion and etching of interdigitated patterns. On comparison with purely pseudocapacitive MSC fabricated through the CMOS compatible process shown in Si et al.,^51^ we note that EDLC materials demonstrate a higher energy and power density in a stacked combination.

For future work in improving MSCs, the pseudocapacitive materials can be tested for spin-coating and stacking using graphene oxide inks. As discussed before, the performance of Stack-MSC resembles the behavior of material with lowest power density, use of pseudocapacitive material would need to be optimized in the carbon material solution. Furthermore, there is a need for investigation in proper packaging techniques for CMOS compatibility for proper encapsulation of ionic liquids. Proper choice of electrolyte is another important consideration for high performance MSCs. MSC performance in devices discussed in the literature survey generally utilize H_2_SO_4_/PVA electrolyte. This is an aqueous electrolyte which has small ions compatible with microporous electrodes. Utilization of H_2_SO_4_/PVA on Stack-MSCs would yield a capacitive performance is expected to be 2–3 times better than EMIM-TFSI. However, using any acid is detrimental to a CMOS fabrication process, as SiO_2_ is reactive to them. Therefore, to ensure CMOS compatibility, we have used an ionic liquid electrolyte. Another direction of work can concern the implementation and advancement of solid state electrolytes with high ionic conductivity.

## Conclusion

In summary, we have presented a heterogeneous stacked multielectrode spin-coated CMOS compatible MSC with four different types of EDLC material based on graphene composites optimized for spin coating. The materials, GO, GO-HD9A, rGO-ODA, and rGO-HD9A, reveal electrochemical and processing qualities such as high capacitance, spin-coated uniformity, and low *R*_*esr*_. We have demonstrated that individual electrode characteristics translate into comparatively high energy density stacked MSCs having a capacitance of 280 μF cm^–2^ while retaining GO’s relatively high power density of 0.1 mW cm^–2^. Hence, we have verified the applicability of a fabrication method that can be used for different materials with controlled spin-coating parameters for the applied electrode solution on a Cr nanoparticle roughened surface for improved adhesion, uniformity, and coverage. The robustness of the fabrication process furthermore enables a more standardized evaluation of electrode material performance for substrate-based planar solid state MSCs.

## Supporting Information Available

Experimental section and characterization information (PDF)
