# Supplementary material for: Spin-Coated Heterogenous Stacked Electrodes for Performance Enhancement in CMOS-Compatible On-Chip Microsupercapacitors
Source: ACS Appl Energy Mater. 2022 Mar 24;5(4):4221–31. doi: 10.1021/acsaem.1c03745 (PMC9044397; doi:10.1021/acsaem.1c03745)
Supplement: Supplementary file 1 — ae1c03745_si_001.pdf [file ae1c03745_si_001.pdf]

Supporting Information

Spin-coated Heterogenous Stacked Electrodes for Performance Enhancement in CMOS compatible On-chip Micro-supercapacitors

Agin Vyas,[*,†] Simin Zare Hajibagher,[†] Ulises Méndez-Romero,[‡] Shameel Thurakkal,[‡] Qi Li,[†] Mazharul Haque,[†] R. K. Azega,[†] Ergang Wang,[‡] Xiaoyan Zhang,[‡] Per Lundgren,[†] Peter Enoksson,[†,¶] and Anderson Smith[§]

[†]Department of Microtechnology and Nanoscience (MC2), Kemivägen 9, 41296, Chalmers University of Technology, Gothenburg, Sweden
[‡]Department of Chemistry and Chemical Engineering, Chalmers University of Technology, Kemigården 4, 41296, Gothenburg, Sweden
[¶]Enoaviatech AB, 112 26 Stockholm, Sweden
[§]Department of Electrical Engineering, Chalmers University of Technology, Hörsalsvägen 7, 41296, Gothenburg, Sweden
[*]E-mail: agin@chalmers.se

S1. Preparation of dispersion

Spin coating requires electrode material in solutions that have good dispersibility. To make graphene based composite material solutions, we started the process by using GNPs and performed the Hummer's process [1] to obtain aqueous solutions of GO. The powder GNP (0.5 g) solution was oxidized in H_2SO_4 (30 ml) and KNO_3 (0.295 g) with $KMnO_4$ (3 g) after six hours of reaction time while being stirred at 450 rpm at a temperature below 15 °C. The solution was quenched with DI-water (100 ml) and H_2O_2 (30 %, 6 ml). The yellow dispersion of GO was centrifuged for 10 min at 3500 rpm with HCl (10 %, 100 ml) added in the precipitate. The precipitate was centrifuged with DI-water three times to obtain a higher purity. The recovered GO is mixed with DI-water and stored as a solution. The functional group HD9A ($(CH_3(CH_2)_7)_2CHNH_2$) are mixed in ethanol and stirred with the GO-solution with solvent at 450 rpm at room temperature for 5 min to achieve GO-HD9A. The GO-HD9A solution is stored and used directly on the wafer substrate for MSC fabrication. Procured GO is mixed with ethanol in presence of ODA and HD9A to obtain GO-ODA and GO-HD9A respectively. Finally, both GO-ODA and GO-HD9A solutions were reduced to rGO using ascorbic acid-6-palmitate (100 ml) at 98 °C for 2 h at 450 rpm. Further information on the synthesis and characterization of the material can be found in the article reported by Mendez et al.[2]. Another GO-H_2O solution

was prepared for spin coating as a control. The details of the solution synthesis can be found in our previous research[3].

S2. Material characterization

The synthesized electrode materials are characterized using Attenuated total reflection-Fourier-transform infrared spectroscopy (ATR-FTIR) for chemical modifications, X-ray photon spectroscopy (XPS) for surface chemical state, and UV-Vis spectroscopy for optical property analysis.

For investigating the chemical modification of the synthesized materials, an ATR-FTIR analysis was performed. To compare the original GO versus the rGOs, the common peaks of oxygen functional groups are presented in Figure 2(a). For instance, the GO spectrum (green line) presents various oxygen functional groups: a typical broadband from 3000 cm^{-1} to 3700 cm^{-1} with a peak at 3200 cm^{-1} that refer to wavenumbers of hydroxyl (OH), carbonyl (C=O) at 1719 cm^{-1}, aromatic (C=C) at 1618 cm^{-1}, alkoxy (C-O) at 1160 cm^{-1} and epoxy (C-O-C) at 1030 cm^{-1}. All features corresponding to highly oxidized material, i.e., graphene oxide, according to previous reports[4]. For rGO-ODA and rGO-HD9A, the prominent hydroxyl band is completely removed, as well as the carbonyl. On the other hand, the most perceptible bands for the two are at 2915 cm^{-1} and 2850 cm^{-1} corresponding to wavenumbers of C-H, respectively [5], confirming a complete chemical reduction process. Furthermore, the vibrations of carbon-nitrogen bonding in the amine (C-NH-C) at 3444 cm^{-1} and at 1560 cm^{-1}, also the wavenumber for NH is recorded at 1470 cm^{-1} [6,7]. The C-O and C-O-C at 1150 cm^{-1} and 1070 cm^{-1} in the rGOs present a small shift from the GO original positions [8]. Finally, N-O bonding can be observed at 718 cm^{-1} and 618 cm^{-1}, respectively [4], suggesting that besides the reduction, the functionalization takes place with the carboxylic and hydroxyl groups. The most interesting feature to distinguish between the ODA and HD9A (linear vs branched alkyl chains), is that transmittance of rGO-HD9A is higher (30 % more), revealing a lower restacking level [9] due to the higher steric hindrance attributed to the branched chains. This feature is relevant for spin coating of rGO as it tends to

agglomerate in the solvent solution after synthesis which can lead to non-uniform electrode deposition.

Furthermore, for a deeper examination of the surface chemical state, the synthesized materials were analyzed by X-ray photoelectron spectroscopy (XPS). The C1s core level spectra are shown in Figure 2(b) for GO, rGO-ODA, and rGO-HD9A. The high-resolution C1s spectra were recorded from 279 eV to 298 eV. The signal coming from the initial material (GNPs – not shown) is composed only by C-C and C=C, with a single contribution at 284.48 eV without any degree of oxidation in accordance with the ATR-FTIR results. After the oxidation of the GNPs by the Hummers' reaction, the C1s signal corresponds not only with C-C but, also C-O and C=O at 286.88 eV and 288.88 eV, respectively [2,10], in agreement with the ATR-FTIR results shown in Fig. 2(a). The signals coming from rGO-ODA and rGO-HD9A are characteristic of highly reduced states, presenting a very small contribution of C-O at 288.08 eV and a new signal related to C-N at 285.48 eV, suggesting a covalent functionalization by the alkyl amine [11].

The analysis of optical band gap reduction (E_g^{opt}) values by UV-Vis spectroscopy is presented in Figure 2(c). The absorbance of rGO-ODA and rGO-HD9A follow a different behavior when compared to GO, i.e., zero absorbance at greater wavelengths than 700 nm. This behavior could be attributed to rGOs because of the linear dispersion by Dirac´s electrons [12–14], which is also good evidence of a highly reduced graphene oxide, with recovered sp^2 hybridization that resembles a more graphene-like material instead of GO.

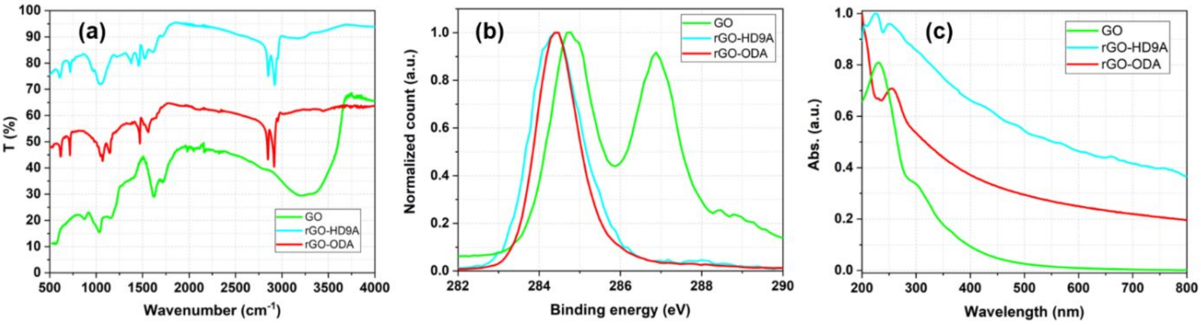

Figure S1: Characterization of synthesized GO based electrodes for spin coated MSCs: (a) ATR-FTIR spectra, (b) XPS C1s survey analysis and (c) UV-Vis-NIR spectra for GO, rGO-ODA, and rGO-HD9A.

To compare the structural quality of the graphene-based materials, Raman spectra (Figure S2) recorded with 2.33 eV laser energy are presented. It is well known that the dispersion of π electrons in graphene offers powerful and efficient insights into their electronic properties, and therefore of their crystallinity. It can be noticed that all spectra exhibit an intense band from 1450-1660 cm^{-1} corresponding to the G band due to vibrational E_{2g} degenerative mode observed in sp^2 carbons. Furthermore, another band is observed at 1260-1400 cm^{-1}, assigned to the D band and related to the A_{1g} mode. The D peak is originated due to the interaction between phonons and defects, such as in-plane substitution heteroatoms, vacancies, or grain boundaries [15–17]. As expected, the material with the best crystallinity is the starting graphite, since it were not subjected to any process. The GO has the lowest crystallinity, but, the rGO-ODA does not recover the crystallinity, despite the highly chemical reduction shown in the XPS results[18–20].

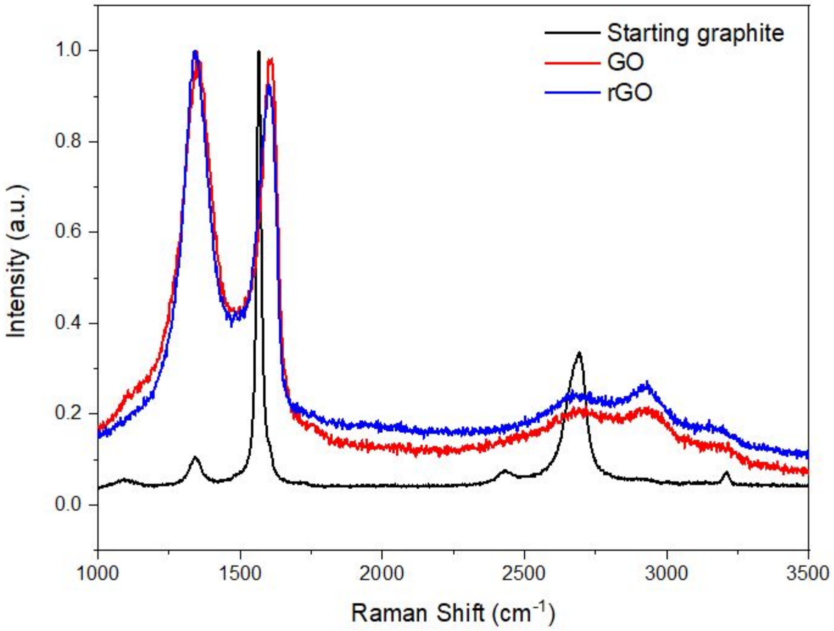

Figure S2: Raman spectroscopy of starting material graphite, GO, rGO

S3. Device characterization

The fabricated MSC wafer contains different types of MSC patterns with different number of fingers, interdigitated spacing, and widths. Each device on the wafer substrate is measured using a Karl Süss PM 5 probe station coupled with a Gamry Reference 3000AE potentiostat. The methodology of device evaluation is kept constant for every device. The device is initially immersed in an EMIM-TFSI electrolyte, an ionic liquid. EMIM-TFSI is chosen as a working electrolyte to establish a realistic performance of MSCs as it demonstrates high charge mobility, thermal stability, and large voltage window. The probe needles are connected to the current collectors and the open circuit potential of the device is measured. The device is then subjected to about 30 cyclic voltammetry measurements at a low scan rate of 20 mV s^{-1} in a 1 V voltage window to achieve a stabilization in performance. Once a constant charging-discharging cycle is obtained, the MSCs are evaluated for cyclic voltammetry measurements at scan-rates of 20 – 5000 mV s^{-1}. The cyclic voltammetric capacitance is calculated using the equation

$$C_x = \frac{Q}{\Delta V x}$$

Where Q is the total stored charge, ΔV is the voltage window, C_x is the capacitance of the device, in which x can be *area* or *volume*. Furthermore, galvanostatic charge-discharge (GCD) measurements were performed at current densities ranging from 0.5 – 10 μA cm^{-2}. The GCD capacitance of MSCs are calculated by checking the drop in electric potential (Δv) when the polarities are switched. We determine the $R_{esr} = \Delta v/(2I)$, equivalent series resistance of the MSC. The capacitance is then calculated as

$$C_x = I\frac{t_d}{(v_f - \Delta v)} \quad (1)$$

where t_d is the discharging time for the MSC, v_f is the maximum final potential, and I is the current. The energy density is calculated as

$$E_x = \frac{1}{2}C_x V^2 \quad (2)$$

V is the voltage window for the MSC. The power density is calculated as

$$P_x = \frac{V^2}{4\,R_{esr}} \ (3)$$

After GCD measurements, we performed electrochemical impedance spectroscopy (EIS) on the devices at a peak-to-peak a.c. signal of 0.5 mV over a range of frequencies ($\omega/2\pi$). The real and imaginary capacitances for a frequency, C_{real} and C_{imag} are calculated as

$$C_{real}(\omega) = \frac{-Z_{imag}(\omega)}{\omega \times |Z(\omega)|^2} (4)$$

$$C_{imag}(\omega) = \frac{Z_{real}(\omega)}{\omega \times |Z(\omega)|^2} (5)$$

Where $\omega = 2\pi f$, Z_{real} and Z_{imag} are real and imaginary impedance of the system respectively. The R_{esr} values from the EIS measurements are calculated by measuring the intercept of the sloping line at the Z_{real} axis. The dielectric time constant, τ is calculated from the peak C_{imag} for a frequency f_0 , also known as the characteristic or knee-point frequency as

$$\tau = \frac{1}{f_0} \ (6)$$

The phase constant of the device (ϕ) is calculated as

$$\phi = \tan^{-1}\left(\frac{Z_{imag}}{Z_{real}}\right) \ (7)$$

Finally, the devices undergo cyclic charge-discharge measurements where the MSCs are initially charged to a potential of 1 V at 5 μA cm^{-2} current density, then the current polarity is switched and the device is discharged to 0 V for 3000 cycles. The device capacitance at the end of each cycle is calculated using equation (2) and then plotted as a function of cycle number. The limitation of the approach is that the tests have been conducted in open atmosphere, therefore, the electrolyte voltage window has not exceeded 1 V as ionic liquids are prone to chemical reactions in air above that potential[21].

References:

1. Hummers, W. S. & Offeman, R. E. Preparation of Graphitic Oxide. *J. Am. Chem. Soc.*

80, 1339 (1958).

2. Méndez-Romero, U. A., Pérez-García, S. A., Xu, X., Wang, E. & Licea-Jiménez, L. Functionalized reduced graphene oxide with tunable band gap and good solubility in organic solvents. *Carbon N. Y.* **146**, 491–502 (2019).

3. Vyas, A. *et al.* Enhanced Electrode Deposition for On-Chip Integrated Micro-Supercapacitors by Controlled Surface Roughening. *ACS Omega* **5**, 5219–5228 (2020).

4. Shimanouchi, T. Tables of molecular vibrational frequencies. Consolidated volume II. *J. Phys. Chem. Ref. Data* **6**, 993–1102 (1977).

5. Pretsch, E., Bühlmann, P. & Badertscher, M. *Structure determination of organic compounds: Tables of spectral data. Structure Determination of Organic Compounds: Tables of Spectral Data* (Springer Berlin Heidelberg, 2009). doi:10.1007/978-3-540-93810-1.

6. Hung, W. S. *et al.* Cross-linking with diamine monomers to prepare composite graphene oxide-framework membranes with varying d-spacing. *Chem. Mater.* **26**, 2983–2990 (2014).

7. Dong, J., Yin, C., Zhao, X., Li, Y. & Zhang, Q. High strength polyimide fibers with functionalized graphene. *Polymer (Guildf).* **54**, 6415–6424 (2013).

8. Acik, M. *et al.* Unusual infrared-absorption mechanism in thermally reduced graphene oxide. *Nat. Mater.* **9**, 840–845 (2010).

9. Nair, R. R. *et al.* Fine structure constant defines visual transparency of graphene. *Science (80-.).* **320**, 1308 (2008).

10. Chua, C. K. & Pumera, M. Light and Atmosphere Affect the Quasi-equilibrium States of Graphite Oxide and Graphene Oxide Powders. *Small* **11**, 1266–1272 (2015).

11. Li, Y. *et al.* Polyimide/graphene composite foam sheets with ultrahigh thermostability for electromagnetic interference shielding. *RSC Adv.* **5**, 24342–24351 (2015).

12. Carey, T. *et al.* Fully inkjet-printed two-dimensional material field-effect heterojunctions for wearable and textile electronics. *Nat. Commun.* **8**, 1–11 (2017).

13. Kumar, P. V. *et al.* Scalable enhancement of graphene oxide properties by thermally driven phase transformation. *Nat. Chem.* **6**, 151–158 (2014).

14. Fernández-Merino, M. J. *et al.* Investigating the influence of surfactants on the stabilization of aqueous reduced graphene oxide dispersions and the characteristics of their composite films. *Carbon N. Y.* **50**, 3184–3194 (2012).

15. Ferrari, A. C. Raman spectroscopy of graphene and graphite: Disorder, electron–phonon coupling, doping and nonadiabatic effects. *Solid State Commun.* **143**, 47–57 (2007).

16. Dresselhaus, M. S., Jorio, A., Hofmann, M., Dresselhaus, G. & Saito, R. Perspectives on Carbon Nanotubes and Graphene Raman Spectroscopy. *Nano Lett.* **10**, 751–758 (2010).

17. Dresselhaus, M. S., Jorio, A. & Saito, R. Characterizing Graphene, Graphite, and Carbon Nanotubes by Raman Spectroscopy. *http://dx.doi.org/10.1146/annurev-conmatphys-070909-103919* **1**, 89–108 (2010).

18. Li, Q. *et al.* Porous graphene paper for supercapacitor applications. *J. Mater. Sci. Technol.* **33**, 793–799 (2017).

19. Li, Q. *et al.* Graphite paper / carbon nanotube composite: a potential supercapacitor electrode for powering microsystem technology. *J. Phys. Conf. Ser.* **922**, 012014 (2017).

20. Brownson, D. A. C., Varey, S. A., Hussain, F., Haigh, S. J. & Banks, C. E. Electrochemical properties of CVD grown pristine graphene: Monolayer- vs. quasi-graphene. *Nanoscale* **6**, 1607–1621 (2014).

21. Dees, A. *et al.* Reactions of Superoxide with Iron Porphyrins in the Bulk and the Near-Surface Region of Ionic Liquids. *Inorg. Chem.* **54**, 6862–6872 (2015).
